# Self-adjuvanted RNActive^® ^vaccines provide a promising platform for combination therapies with checkpoint inhibitors

**DOI:** 10.1186/2051-1426-2-S3-P109

**Published:** 2014-11-06

**Authors:** Aleksandra Kowalczyk, Stephan Huber, Regina Heidenreich, Mariola Fotin-Mleczek

**Affiliations:** 1 CureVac GmbH, Tuebingen, Germany; 2 Dept. of Radiooncology, Tuebingen, Germany

## 

Two-component mRNA vaccines (RNActive^®^) combine high antigen expression with strong immune stimulation. Intradermal injections of RNActive^® ^vaccines induced balanced, potent and long-lasting immune responses which can be boosted via repetitive vaccinations.

Although the strong immune response induced by RNActive^® ^vaccination alone already led to a potent therapeutic anti-tumor response in the E.G7-Ovalbumin tumor model, the combination of RNActive^® ^vaccination with radiation or checkpoint inhibitors elicited strong synergistic effects, resulting in an even more effective anti-tumor response.

Monotherapy with either OVA-RNActive^® ^or radiation was not effective in curing mice bearing large E.G7-OVA tumors. Also, high dose radiation of tumors induced only transient growth stagnation. In contrast, the combination of RNActive^® ^vaccination and radiation dramatically improved anti-tumor efficacy and supported surveillance of large tumors in the treated mice.

Combinatorial treatment of mice bearing already established E.G7-OVA tumors with OVA- RNActive^® ^and checkpoint inhibitors such as anti-CTLA-4 or anti-PD-1 monoclonal antibody resulted in a strong, synergistic anti-tumor effect, yielding a higher frequency of mice with complete tumor rejection compared to treatment with the single components. Remarkably, these complete responders were protected against re-challenge with parental ovalbumin-negative EL-4 tumors, indicating antigen spreading in these groups.

The safety and immunogenicity of RNActive^® ^vaccines have been already successfully tested in humans in two cancer indications: non-small cell lung cancer (CV9201) and prostate cancer (CV9103). Data from both trials revealed induction of the immune responses against all antigens included in the vaccination protocol.

In conclusion, our results demonstrate that intradermal vaccinations with self-adjuvanting RNActive^® ^induce potent anti-tumor immune responses which can be further enhanced by combination with other therapies such as checkpoint inhibitors or radiation. Thus, RNActive^® ^vaccines constitute an attractive vaccination platform for combination therapies in the field of cancer immunotherapy.

